# Epidemiology of road traffic crashes in Ilam Province, Iran, 2009–2013

**DOI:** 10.1186/s13104-020-05366-x

**Published:** 2020-11-10

**Authors:** Marzieh Mansouri Jalilian, Hamid Safarpour, Jafar Bazyar, Meysam Safi-Keykaleh, Iman Farahi-Ashtiani, Ali Khorshidi

**Affiliations:** 1 Psychosocial Injuries Research Center, Ilam University of Medical Sciences, Ilam, Iran; 2 Department of Nursing, Faculty of Nursing and Midwifery, Ilam University of Medical Sciences, Ilam, Iran; 3 Department of Health in Disasters and Emergencies, School of Public Health and Safety, Shahid Beheshti University of Medical Sciences, Tehran, Iran; 4 Malayer School of Nursing, Hamadan University of Medical Sciences, Hamadan, Iran; 5 Department of Epidemiology, School of Medicine, Ilam University of Medical Sciences, Ilam, Iran

**Keywords:** Road safety, Road traffic injury, Risk factors, Vehicle, Accident, Trauma

## Abstract

**Objective:**

Road traffic crashes (RTCs) are major public health challenges of world health systems, and the main leading cause of death in children and young adults aged 5 to 29 years. This study aimed to assess the epidemiology of RTCs in Ilam, Iran.

**Results:**

The total mortality rate due to RTCs has been increasing every year. There was a statistically significant relationship between age/sex and death or injury from RTCs in pedestrians, drivers, and passengers (p < 0.05). There was a significant relationship between the part of body trauma and RTCs in pedestrians (p < 0.001). Furthermore, a significant relationship was found between the type of vehicle and using seat belt with RTCs in drivers and passengers (p = 0.000).

## Introduction

Road traffic crashes (RTCs) are the principal public health problem in developed and developing countries [1–3]. RTCs are defined as fatal or non-fatal injuries incurred as a result of a road traffic crash [4]. Death from RTCs affect not only victims, but also families and the wider community at the national level with physical, psychological, economic, and quality of life consequences [5]. Approximately, 1.35 million people die each year as a result of RTCs [6]. More than 90% of deaths occur in low and middle-income countries [7]. They are the main leading cause of death for children and young adults aged 5 to 29 years [6]. The causes of road crashes can be attributed to human, vehicular, and environmental factors. Besides the environmental factors, Geographical conditions and climate change can increase the number of RTCs [8]. Moreover, Temperature, rainfall, sandstorms, and number of vehicles are responsible for RTCs [9].

Iran has one of the highest mortality rates from RTCs among middle-income countries [10, 11], which are the leading cause of early death and years of life lost (YLL), and the most common cause of injuries [12, 13]. Approximately, 0.8 million people (1.1% of Iran’s population) are hospitalized per year due to RTCs, which imposes a significant burden on the country's health system. Also, more than one-third of hospital beds are allocated to RTCs victims [14]. In a study conducted between 2004 and 2011, the average rate of deaths from RTCs was 31 per 100,000 population [15]. Also, according to the WHO report in 2015, the mortality rate caused by RTCs in Iran was 32 deaths per 100,000 persons [12].

In Ilam province of Iran, due to the climatic and geographical conditions, RTCs result in major life and financial losses annually [16]. The rate of death due to RTCs in Ilam province in 2012 was estimated to 24/82 per 100,000 people. Furthermore, as an strategic location, this province is exposed to high road traffic for about 30 days every year in Arbaeen mass gathering [17, 18]. At this time, travel to the border is by road and personal vehicles, which increases the risk of RTCs [17]. Therefore, prevention of these injuries is necessary and prevention clearly requires recognition and attention to the pattern of risk factors. Knowing the intervention with more impact on RTCs mortality prevention by authorities may result in better planning to prevent the consequences of these types of events in the province at a lower cost and higher efficiency. Due to the lack of comprehensive studies on the epidemiology of RTCs in Ilam province, this study aimed to assess the epidemiology of RTCs in Ilam, Iran.

## Main text

### Methods

This was a retrospective cross-sectional study. Data on RTCs in Ilam were gathered during a five-years period (2009–2013) using census method. The data were collected from COM114 police form filled by road safety experts at the accident scene. This form contains a relatively large number of variables that are used by the police in all traffic accidents. Data are collected in two parts of general and specific information. In the general section, information about time, place, road conditions, weather conditions, lighting conditions, vehicle characteristics, characteristics of people (driver, pedestrian and passenger) and causes of the crashes are collected. In the specific section, information related to crash maps and expert advice are collected and entered in the traffic crashes database. The tool questions are designed to be open and closed. The data is entered into the system by the police in different parts of the country and it is possible for users to process and report it at the local and national levels. Permission was obtained from Ilam University of Medical Sciences and approved by the ethical committee. The aims of the study were explained to the police office. Then, for the retrieval of individual records and confidentiality of information, written consent was given to the record office of the police. The collected information was about the environmental conditions of injuries, human factors, and vehicle characteristics as well as the consequences of RTCs. Data in the police database is maintained in four separate sections and stored in the Microsoft Excel®. The data of each section were reviewed separately and controlled and modified in terms of human errors (redundancy, typos errors, etc.) as necessary. To use all the data related to a specific crash, the data of these four sections were linked through common keywords. The method of controlling missing data was to add them to the categories that had the highest frequency. The missed data were considered as variables that have a more frequent rate than others. Data were entered into the SPSS-20 software and examined in three categories of pedestrians, drivers, and passengers. In each category, the relationship of the dependent variable (injury or death due to RTCs) with independent variables using Chi-square test, as well as the incidence rate of death or injury in terms of sex and age during the studied years were analyzed.

### Results

During the five-year period, the crude mortality rate has been increasing every year from 22.06 in 2009 to 43.23 in 2013, per 100,000 population. The mean age-standardized incidence rate was 23.03 per 100,000 population. The rate of death from RTCs in passengers increased every year from 5 in 2009 to 22 in 2013 per 100,000 population.

There was a statistically significant relationship between age and death or injury from RTCs in all the three categories of pedestrians, drivers and passengers (P < 0.05). The number of deaths in males in all the three categories of pedestrians (40, 2.8%), passengers (299, 8.9%), and drivers (462, 2.2%) was more than those of females. There was a significant relationship between sex and death or injury from RTCs in pedestrians, passengers and drivers (P < 0.05). In all the categories, the deaths and injuries in males were more than those of females. Most pedestrians (708, 80%) had an educational level under a high school diploma, and none of the pedestrians with academic education died. There was a significant relationship between education level and death or injury from a RTCs in drivers and passengers (P < 0.05). The people with Non-Academic education had the highest rate of death or injury.

There was a significant relationship between employment type and death or injury from RTCs in drivers. Employed drivers had higher rate of deaths or injuries compared to unemployed drivers (P = 0.05). However, there was no statistically significant relationship between employment type and death or injury in pedestrians and passengers (Table 1). A significant relationship was found between the type of vehicle and the RTCs in drivers (P < 0.001). Most drivers (78.3%) who had an accident with a small vehicle were injured, and 69 (0.3%) of the drivers who had an accident with a motorcycle have died.

**Table 1:** Frequency distribution of RTCs in pedestrians, passengers and drivers by demographic characteristics

Variable	Pedestrian			Driver			Passenger		
	Injured	Died	Total	Injured	Died	Total	Injured	Died	Total
Age(year)									
20 <	376 (26.9%)	15 (1.1%)	391 (28%)	1208 (6.1%)	34 (2%)	1242 (6.3%)	517 (21.6%)	84 (3.5%)	601 (25.1%)
21–40	469 (33.5%)	6 (4%)	475 (34%)	13,430 (67.9%)	264 (1.3%)	13,694 (69.3%)	1148 (47.9%)	148 (6.2%)	1296 (54.1%)
41–60	302 (21.6%)	14 (1%)	316 (22.6%)	4407 (22.3%)	112 (6%)	4519 (22.9%)	343 (14.3%)	72 (3%)	415 (17.3%)
> 60	206 (14.7%)	10 (7%)	216 (15.5%)	307 (1.6%)	7 (0%)	314 (1.6%)	70 (2.9%)	14 (6%)	84 (3.5%)
Total	1353 (96.8%)	45 (3.2%)	1398 (100%)	19,352 (97.9%)	417 (2.1%)	19,769 (100%)	2078 (86.7%)	318 (13.3%)	2396 (100%)
P value	0.02			0.05			0.01		
Sex									
Female	521 (36.1%)	9 (0.6%)	530 (36.7%)	504 (2.3%)	2 (0%)	506 (2.4%)	1209 (35.9%)	158 (4.7%)	1367 (40.6%)
Male	873 (60.5%)	40 (2.8%)	913 (63.3%)	20,511 (95.5%)	462 (2.2%)	20,973 (97.6%)	1700 (50.5%)	299 (8.9%)	1999 (59.4%)
Total	1394 (96.6%)	49 (3.4%)	1443 (10%)	21,015 (98.7%0	464 (2.2%)	21,479 (10%)	2909 (86.4%)	457 (13.6%)	3366 (100%)
P value	0.007			0.006			0.005		
Educational level									
Illiterate	129 (14.6%)	7 (8%)	136 (15.4%)	441 (4%)	9 (1%)	450 (4.1%)	142 (6.9%)	26 (1.3%)	168 (8.1%)
Non-Academic	708 (80%)	22 (2.5%)	730 (82.5%)	9187 (83.1%)	150 (1.4%)	9337 (84.4%)	1642 (79.4%)	208 (10.1%)	1850 (89.5%)
Academic	19 (2.1%)	0 (0%)	19 (2.1%)	1267 (11.5%)	8 (1%)	1275 (11.5%)	39 (1.9%)	10 (5%)	49 (2.4%)
Total	856 (96.7%)	29 (3.3.%)	885 (100%)	10,895 (98.5%)	167 (1.5%)	11,062 (100%)	1823 (88.2%)	244 (11.8%)	2067 (100%)
P Value	0.31			0.01			0.04		
Employment type									
Unemployed	153 (28.8%)	2 (0.4%)	155 (29.1%)	2290 (18.2%)	18 (1%)	2308 (18.4%)	161 (33.4%)	15 (3.1%)	176 (36.5%)
Employed	371 (69.7%)	6 (1.1%)	377 (70.9%)	10,115 (80.6%)	129 (1%)	10,244 (81.6%)	291 (60.4%)	15 (3.1%)	306 (63.6%)
Total	524 (98.5%)	8 (1.5%)	532 (100%)	12,405 (98.8%)	147 (1.2%)	12,552 (100%)	452 (93.8%)	30 (6.2%)	482 (100%)
P Value	0.79			0.05			0.11		

There was a statistically significant relationship between using seat belts and death or injury from RTCs in drivers and passengers (P < 0.001). Most drivers (3819, 65.7%) who used seat belts were injured and 122 (1.2%) who had not used seat belts died. A total of 123 (11%) of passengers that did not fasten their seat belts died. There was a significant relationship between the type of motor vehicle and death or injury in drivers and passengers (P = 0.000). Light vehicle drivers have more deaths and injuries compared to heavy vehicles and motorcycles (Table 2).

**Table 2:** Type of crash by type of vehicle, and seatbelt by driver and passenger

Variable	Driver			Passenger		
	Injured	Died	Total	Injured	Died	Total
Motor vehicle						
Heavy	2395 (11.1%)	132 (0.6%)	2527 (11.7%)	128 (3.8%)	114 (3.4%)	242 (7.2%)
Light	16813 (78.3%)	266 (1.2%)	17079 (79.5%)	2402 (71.4%)	310 (9.2%)	2712 (80.7%)
Motorcycle	1806 (8.4%)	69 (0.3%)	1875 (8.7%)	375 (11.2%)	33 (1%)	408 (12.1%)
Total	21014 (97.8%)	467 (2.2%)	21481 (100%)	2905 (86.4%)	457 (13.6%)	3362 (100%)
P value	0.000			0.000		
Seat belt						
Used	3819 (65.9%)	133 (2.3%)	3952 (68%)	297 (26.5%)	19 (1.7%)	316 (28.2%)
Not used	1740( 29.9%)	122 (2.1%)	1862 (32%)	680 (60.8%)	123 (11%)	803 (71.8%)
Total	5559 (95.6%)	255 (4.4%)	5814 (100%)	977 (87.3%)	142 (12.7%)	1119 (100%)
P value	0.000			0.000		

Part of body trauma had a statistically significant relationship with death or injury from RTCs in pedestrians (P < 0.001), while there was no such relationship in passengers. Most pedestrians (37, 80%) and passengers (1977, 76.66%) were injured in the head and face, and none of the pedestrians who were injured in the lower extremities died. (Table 3).

**Table 3:** Part of body trauma and RTCs in pedestrian and passenger

Variable	Pedestrian			Passenger		
	Injured	Died	Total	Injured	Died	Total
Part of body						
Head and face	375 (80%)	4 (1%)	379 (81%)	1977 (76.6%)	327 (12.6%)	2304 (89.26%)
Upper limbs	56 (12%)	5 (1%)	61 (13%)	175 (6.78%)	13 (0.005%)	188 (7.28%)
Lower limbs	29 (6%)	0 (0%)	29 (6%)	82 (3.17%)	7 (0.002%)	89 (3.44%)
Total	460 (98%)	9 (2%)	469 (100%)	2234 (86.55%)	347 (13.44%)	2581 (100%)
P value	0.000			0.22		

### Discussion

In this study, the rate of death from RTCs in the province of Ilam has been increasing, which might be due to the higher number of vehicles and more exposure of people. This rate (43.23) was higher than the mean rate in the Eastern Mediterranean region (32.2) [19]. It was also higher than the Iran mean rate of 31 per 100,000 population in a study by Bahadory Monfared et al. in Iran [15], and far more than the rate of 33.4 per 100,000 population in a study by Entezami et al. in northern provinces of Iran. It might be due to the specific climatic and geographical conditions of the province of Ilam [20]. However, it was lower than 52.9 per 100,000 population in a study by Hasani et al. in Semnan, Iran [21].

The results showed a significant relationship between age and death or injury from RTCs. Some studies show that there was no significant relationship between age and death or injury, which was not consistent with this study [22, 23]. However, other studies show that age has a significant relationship with death or injury from RTCs [11, 24]. Elderly pedestrians had a greater risk of death compared to younger individuals [25]. It may be a result of other confounding risk factors for pedestrian injury outcome rather than a positive relationship between pedestrian age and severity of the outcome [26].

In this study, a significant relationship was found between sex and death or injury from RTCs. The results of some studies conducted in the field of RTCs and their epidemiology of injury or death showed that sex had the greatest effect on placing people in the injury or death groups, which was consistent with this study [11, 12, 24, 25, 27, 28]. However, a study that assessed “the influence of age on the morbidity and mortality of pedestrian victims” showed that there was no relationship between sex and injury severity or death [26]. International studies reveal that the male-to-female ratio of fatal RTCs worldwide is 2.7:1 [29], which was lower than the one observed in the study 5.6/1 [30]. This gender difference can be attributed to the increased susceptibility and exposure of men due to specific occupational, cultural, and social issues [12]. Also, it is maybe due to more exposure and more dangerous behaviors of males in contrast to the more cautious behaviors of females during driving [28].

A significant relationship was found between education level and death or injury from RTCs in drivers and passengers. Most pedestrians had an educational level under a high school diploma, and none of the pedestrians with had academic education died. The results of the present study were similar to the results of previous studies [23, 30–32]. One study shows that one-fifth of the deaths from road traffic crashes occurred among illiterate people and people with university-level education have the lowest proportion [30]. A significant effect of education level status on traffic accident death tolls was observed [31, 32], and with higher education level, the injury incidence reduced [23]. This result is partially explained as drivers with higher educational level have better economic and cultural status enabling them to own more expensive and standard vehicles as well as more respect to the driving regulations [12].

In this study, no relationship was found between the employment type of drivers and death or injury from RTCs. Moreover, there was no statistically significant relationship between employment type and death or injury in pedestrians and passengers. In a study of the relationship between injury and socioeconomic status, the results showed no significant relationship between employment type and injury from road crashes [23].

There was a significant relationship between the type of motor vehicle and death or injury in drivers and passengers (P = 0.000). A study on RTCs’ consequences from different types of road user showed that motorcyclists and pedestrians suffer the most severe injuries and more complicated medical problems [33]. Based on the findings of a study, due to force or the sharp parts of vehicles, RTCs with a specially equipped vehicle such as oversize loaded trucks is more injurious than other vehicle types including trucks, buses and sedans [33].

The results indicated that the use of seat belts had a significant relationship with traffic crashes leading to death or injury, and findings from previous studies confirmed this [34, 35]. The use of seat belts is compulsory in Iran, but most people do not use this safety tool [36]. It reduces the damage by preventing the occupants from colliding with the interior of the vehicle or being thrown out of the vehicle [37]. Although seat belt has been proven to decrease the severity of injury as well as mortality, it is also associated with specific injury patterns [38]. Trauma to the head and face was the most common cause of death in pedestrians and passengers in this study. Many studies show that the most common cause of injury or death from RTCs was a strike in the head, which was consistent with this study [24, 25, 28, 36, 39, 40].

### Conclusion

It appears that policies and educational programs for young and middle-aged groups will contribute to reducing deaths from RTCs. Also, requiring passengers to comply with driving regulations and fastening seat belts appear to be necessary to prevent and minimize death from road traffic crashes. Reducing mortalities due to RTCs should be the top priority for the government and health-care system. A multistage approach to injury-related problems and death and the focus on the groups who need priority intervention are necessary to reduce the incidence of injury and death from RTCs.

## Limitations

This study was limited to the data collected from the COM 114 form, which were incomplete because they were not collected for research purposes.

## Data Availability

All data obtained during this study is included in this article. The datasets used and/or analysed during the current study are available from the corresponding author on reasonable request.

## Abbreviation

RTCs: Road Traffic Crashes
